# α-NETA down-regulates CMKLR1 mRNA expression in ileum and prevents body weight gains collaborating with ERK inhibitor PD98059 in turn to alleviate hepatic steatosis in HFD-induced obese mice but no impact on ileal mucosal integrity and steatohepatitis progression

**DOI:** 10.1186/s12902-023-01267-9

**Published:** 2023-01-10

**Authors:** Canbin Zheng, Yongping Zheng, Xi Chen, Xianyang Zhong, Xiaobin Zheng, Shuhui Yang, Zihui Zheng

**Affiliations:** 1 Department of Endocrine and Metabolic Diseases, Shantou Central Hospital, Shantou, Guangdong China; 2 Department of Gastroenterology, Shantou Central Hospital, 114 Waima Road, Shantou, 515031 Guangdong China; 3 Department of Clinical Medicine Research Center, Shantou Central Hospital, Shantou, Guangdong China

**Keywords:** Chemerin/Chemokine-like receptor-1 (CMKLR1), 2-(α-naphthoyl) ethyltrimethylammonium iodide (α-NETA), Extracellular-regulated kinase (ERK), Western blotting; rtPCR, Zonula occluden-1(ZO-1)

## Abstract

**Background:**

Studies on chemerin/chemokine-like receptor-1 have mainly focused on adipose and liver with the intestinal tissues largely overlooked. In this study conducted on obese mice, we have explored: 1) CMKLR1 expression in the ileums; 2) CMKLR1 inhibitor α-NETA on body weight and intestinal mucosa integrity hence the impact on hepatic steatosis and pathway involved.

**Methods:**

Nineteen male C57BL/6 mice were randomly divided into five groups: normal diet group (ND), high-fat diet group (HFD), HFD + α-NETA group (NETA), HFD + PD98059 group (PD) and HFD + α-NETA + PD98059 group (NETA + PD). Mice were fed either with a chow diet or HFD for 12 weeks. At 12^th^ week, mice of ND were put on the diet as before; mice of NETA received daily treatments of α-NETA (30 mg/kg) via gavage; mice of PD received daily treatment of PD98059 via tail vein injection; mice of NETA + PD received daily treatment of α-NETA + PD98059, all for another 4 weeks. At the time intervention ended, mice were sacrificed. The body weight, the liver pathologies were assessed. Ileal CMKLR1 mRNA was evaluated by rtPCR; ZO-1, ERK1/2 protein expression of ileal tissues by western blotting; liver TNF-α and serum endotoxin by Elisa.

**Results:**

More weight gains in mice of HFD than ND (37.90 ± 3.00 g) vs (24.47 ± 0.50 g), *P* = 0.002; α-NETA reduced the body weight (33.22 ± 1.90 g) vs (37.90 ± 3.00 g), *P* = 0.033; and further reduced by NETA + PD98059: (31.20 ± 1.74 g) vs (37.30 ± 4.05 g), *P* = 0.032. CMKLR1 mRNA expression was up-regulated in ileum in group HFD compared with ND and down-regulated by α-NETA. Steatosis was only alleviated in group PD + NETA with less weight gain. No impact of α-NETA on ileal ZO-1 or pERK with western blotting, and no endotoxin level changes were detected. TNF-α was higher in group HFD than in group ND, while no significant difference between other groups.

**Conclusions:**

CMKLR1 mRNA was up-regulated in the ileum of obese mice and down-regulated by α-NETA along with a body weight control collaborating with ERK inhibitor PD98059. Steatosis was alleviated in a weight dependent way. α-NETA has no influence on intestinal mucosal integrity and no impact on steatohepatitis progression.

**Supplementary Information:**

The online version contains supplementary material available at 10.1186/s12902-023-01267-9.

## Background

Obese and Non-alcoholic fatty liver disease (NAFLD) are fast emerging as global health problem. Factors such as insulin resistance, increased free fatty acids, and changes in cytokine levels accompanying obesity exert a significant influence in the process of NAFLD [1]. The etiology of NAFLD remains mostly unclear although the two-hit and multi-hits theory has been brought up. In the two-hit theory, the first hit is the accumulation of triglycerides (TG) in hepatocytes while the second-hit is the hepatocellular injury, inflammation and fibrosis caused by inflammatory mediators. The multi-hits model mainly focuses on fatty acids and the metabolites. It is believed that insulin resistance enhances the hepatic deposition of free acids, further activates endoplasmic reticulum, oxidative stress and ultimately apoptosis of hepatocytes, in turn to facilitate the development of simple steatosis to steatohepatitis [2–4]. The intestinal epithelium constitutes a barrier that separates the host from the food and microbiota in the gut. Fat in the daily diet, together with other factors, may cause gut microbiota dysbiosis, and alterations in intestinal permeability, thus resulting in NAFLD. Studies have also shown that gut microbiota and their products accelerate the progress of NAFLD [5]. Hence, much effort has been adopted regarding changes in the intestinal epithelium in response to HFD ingestion, both in the small intestine and colon.

Chemerin is an adipokine related to inflammation, immunity, and metabolism [6]. Studies have demonstrated that chemerin/CMKLR1 signaling plays an essential role in the recruitment of CMKLR1expressing cells to sites of localized inflammation or tissue damage and also correlated with development of NAFLD [7–9].

The expression of CMKLR1 in steatotic liver tissues have been reported but with conflicting results. Some believe the upregulated CMKLR1 alleviated steatosis [10] while others revealed the opposite findings [11, 12]. The correlation of CMKLR1 with body weight control was reported by Ernst et al. [13], who demonstrated that CMKLR1-knockout mice have reduced weight compared to wild-type when fed on either a low- or a high-fat diet. We believe CMKLR1 may have due effects and may be influenced by liver functions, we hypothesized that changes of CMKLR1 on intestinal tissues may do help to get insight its roles.

In the present study, we explore the protein and mRNA of CMKLR1 in mice ileum; investigate the effect of α-NETA, an inhibitor of CMKLR1, on modulating weight gains of HFD obese steatotic mice. Based on the report that chemerin is highly expressed in white adipose tissue, liver and lung while its receptor CMKLR1 is predominantly expressed in adipocyte [14], and the study revealing that the brown adipose tissue thermogenesis alleviates obesity by increasing energy expenditure which is regulated by MAPK signaling [15]. We also applied PD98059, an inhibitor of ERK pathway to investigate whether ERK pathway is involved in its effects. Since study has revealed that gut microbiota dysbiosis, and alterations in intestinal permeability, may be related with NAFLD [16]. And the increasing expression of ZO-1, which was observed in epidermal tight junctions, has been believed to rebuild the tissue structure of intestinal mucosal epithelial cells [17]. The impact of α-NETA on ZO-1 and endotoxin, which represent the intestinal mucosal integrity, was also studied.

## Methods

### Animals and experimental design

24 wild-type adult C57BL/6 mice (male, 8-10 weeks old, 18-22 g) were obtained from Jicui Yaokang biotech co. Mice received either a standard diet high-fat high-fructose (HFD, 40 kcal% fat, 2% fructose, Moldiet, China) for 15 weeks after initiating the diet. Water was freely available at all times. Mice were housed at 23 ± 1 °C with an average humidity of 60 ± 1% and a 12-h light/dark cycle. The body weights were measured weekly.

After one week of acclimatization, mice were randomly assigned to the following 5 groups: normal diet fed group (group ND), high-fat diet fed group (group HFD), HFD plus α-NETA treated groups (group NETA), HFD plus PD98059 group (group PD) and HFD plus α-NETA plus PD98059 groups (group NETA + PD). Group ND and group HFD received solvent control; Group NETA received daily treatments of α-NETA (30 mg/kg) via gavage. Group PD received daily treatment of PD98059 (0.3 mg/kg) via tail vein injection. Group NETA + PD received daily treatment of α-NETA plus PD98059. The dose of α-NETA was referred to previous published work by kareem, et al. [18]. α-NETA and PD98059 were purchased from MedChemExpress. These treatments were beginning at the 12^th^ week after disease induction. Experiment protocols were approved by ethics committee of Shantou Central Hospital. Mice received humane care and the study is reported in accordance with ARRIVE guidelines.

Mice were anesthetized and sacrificed with isoflurane. Livers were harvested at the end of the experiment, weighted and immediately placed in 4% paraformaldehyde for 12 h. The contents of the intestinal cavity were washed with ice-cold physiological saline. A 05–1 cm length of ileum 2–3 cm away from the ileocecal junction was removed and frozen in liquid nitrogen or treated with RNA store solution (Tiangen, China), then stored at -80 °C for RNA or protein analysis.

### Liver histology

Liver were dehydrated and embedded in paraffin after 4% paraformaldehyde treatment. Hematoxylin and eosin ((H&E) were performed with 4 μm thick paraffin sections following the standard protocol. Investigators were blinded to the group identity of each section.

Slices were classified into four categories depending on fat accumulation using a previously established method which defines the degree of steatosis graded 0–4 according to magnitude of steatosis [19].

### RNA analysis

Total RNA was extracted using TRIzol® Reagent (Invitrogen, Carlsbad, CA, USA) according to the manufacturer’s protocol. Ileal tissues were homogenization using Tissue Lyser (Beyotime, China). Reverse transcription polymerase chain reaction was carried out using Applied Biosystems 7500 PCR system (Carlsbad, CA, USA). Total RNA was used as a template for first strand cDNA synthesis using PrimerScrip RT Master Mix Kit (Accurate Biology, China). The primer set used in this study were as follows. CMKLR1: 5'-CATCGTCTTCAAGTTGCAGC-3’ and 5’-AGCAGGTAGAGTGTGTGGTAGG-3’. GAPDH (as internal control): 5’-GCT GAGTATGTCGTGGAG-3’ and 5’-TCTTCTGAGTGGCAGTGAT-3’. The 2 − ΔΔCt method was applied to calculate the fold change of relative gene expression.

### Western blotting analysis

Proteins were extracted from ileal tissues using RIPA lysis buffer (Beyotime, China) with 1% PMSF (Beyotime, China). Protein content of the samples was measured by BCA Protein Assay Kit. Proteins (40 μg per sample) were separated by SDS-PAGE with 8% or 10% polyacrylamide gels and transferred to PVDF membranes. The blots were blocked with a solution of 5% skim milk in TBST for 1 h at room temperature and in incubated overnight at 4 °C with primary antibodies (CMKLR1, AF5291, Affinity Bioscience; ZO-1, sc33725, Santa cruz; pERK 1/2, sc81492, Santa cruz, CA, USA; β-tubulin, sc166729, Santa cruz; GAPDH, AB0037, Abways). Membranes were incubated with respective secondary antibodies (goat anti-mouse IgG (H + L), BA1050, BOSTER; rabbit anti-rat IgG (H + L), BA1058, BOSTER; goat anti-rabbit IgG (H + L), AB0101, Abways for 1 h at room temperature. ZO-1, pERK1/2 and GAPDH proteins were detected by SuperSignalÔ west pico PLUS chemiluminescent substrate (Thermo, USA) and ChemiDocÔ imaging system (Bio-Rad, USA). The integrated intensity for the protein bands was determined by ImageJ software (NIH, Bethesda, Maryland, USA) and was analyzed using relative intensity to the constitutive marker, GAPDH or β-tubulin.

### Biochemical determinations and endotoxin detection

Serum levels of alanine aminotransferase (ALT), aspartate aminotransferase (AST), total cholesterol (TC), and triglycerides (TGs) were measured with an automatic bio-chemical analyzer. Endotoxin was determined using ELISA kits (Bioendo, Xiamen, China) according to the manufacturer's protocols.

### Statistical analysis

Statistical analysis of results was performed using SPSS version 23. Values are expressed as the mean ± standard deviation. An unpaired *t*-test was used to compare data between groups. A value of *P* < 0.05 was considered statistically significant.

## Results

### Characteristics of animals

A total of 24 mice were investigated in the study, 5 mice died in the experiment procedure, 19 mice were finally analyzed. The initial body weights of the mice were similar (23.15 ± 1.00 g).

### α-NETA reduced body weight gain collaborating with PD98059

**Fig. 1:**
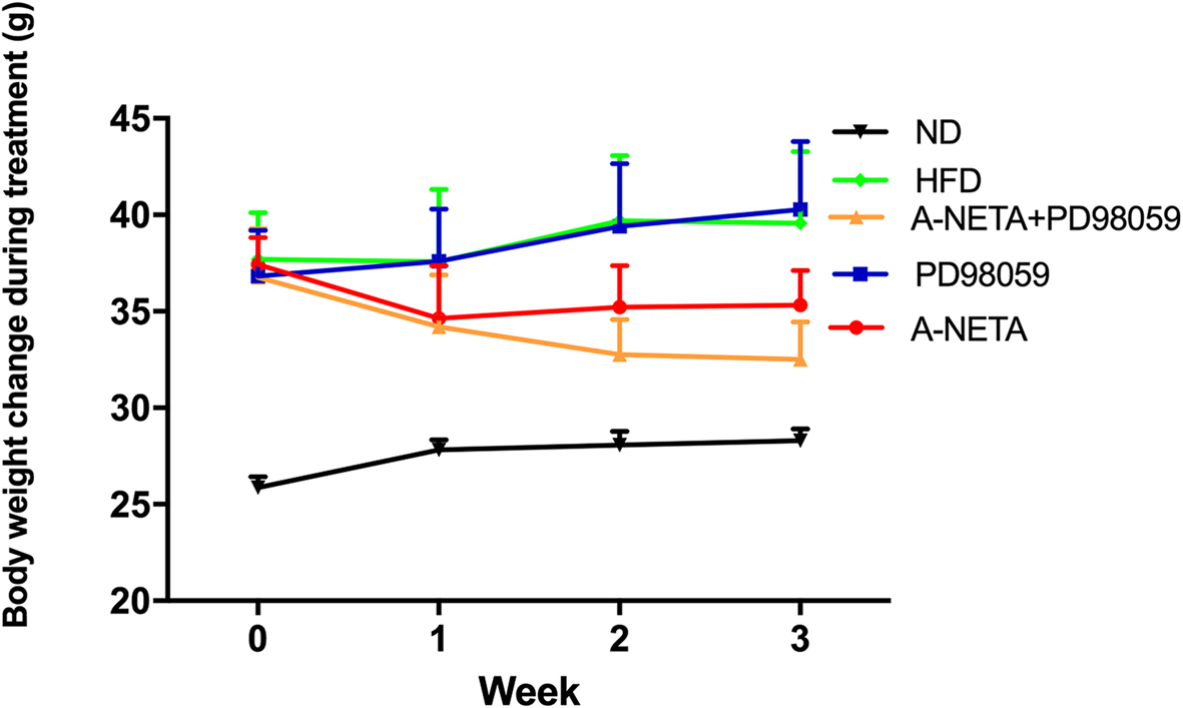
shows α-NETA reduced the body weight, and the body weights further decreased in group NETA + PD, with significant differences compared with group HFD

No difference in mean weight was observed between groups of ND, HFD, NETA, PD and NETA + PD at the time intervention started. At the time intervention ended, data showed more body weight in group HFD than in group ND: (37.90 ± 3.00 g) vs (24.47 ± 0.50 g) *P* = 0.002; α-NETA reduced the body weight: (33.22 ± 1.90 g) vs (37.90 ± 3.00 g) *P* = 0.033; and further decreased in group NETA + PD: (31.20 ± 1.74 g) vs (37.30 ± 4.05 g) *P* = 0.032, indicating a synergic effect of α-NETA and PD98059 in modulating body weights (Fig. 1, Table 1).

**Table 1:** Scores of steatosis, body weight, liver TNF-α and serum endotoxin in different groups

Group	Body weight	Score of steatosis	TNF-α (pg/mg)	Endotoxin (EU/ml)
HFD	37.90 ± 3.00	3.78 ± 0.44	3.04 ± 0.12	0.55 ± 0.48
NETA	33.22 ± 1.9 ^a^	3.50 ± 0.65	2.74 ± 0.40	0.07 ± 0.22
PD	37.30 ± 4.05	3.90 ± 0.32	2.10 ± 0.74	-0.02 ± 0.05
PD + NETA	31.20 ± 1.74 ^a^	2.73 ± 0.65^a^	2.38 ± 0.73	-0.00 ± 0.08
ND	24.47 ± 0.50 ^b^	0 ± 0	1.40 ± 0.20^c^	0.04 ± 0.12

^a^*P* < 0.05 vs group HFD

^b^*P* < 0.01 vs group HFD

^c^*P* < 0.001 vs group HFD

### α-NETA and α-NETA plus PD98059 improved hepatic steatosis but no impact on TNF-α level of liver homogenates

**Fig. 2:**
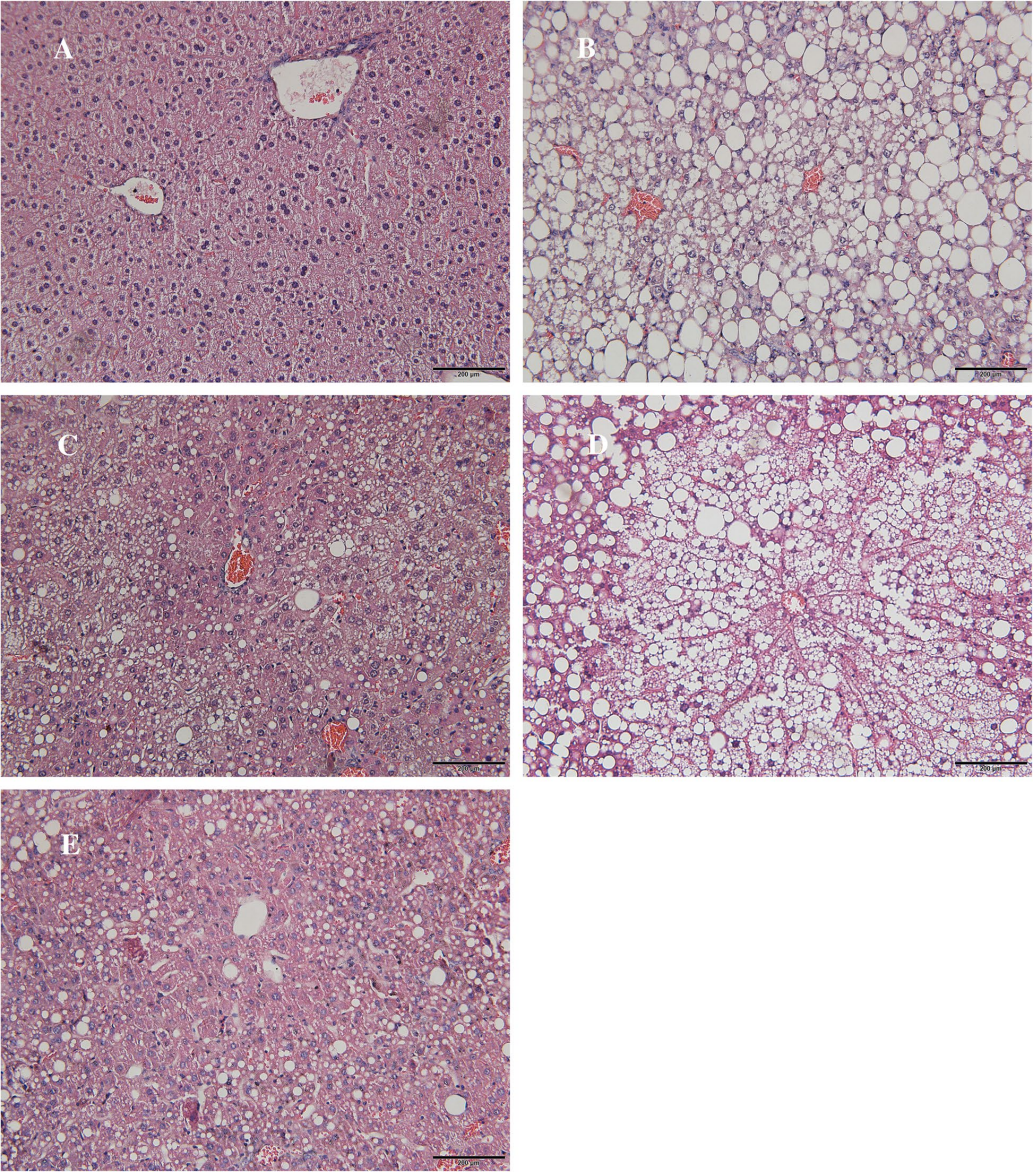
**A** to **E** shows steatosis is less severe in group of NETA + PD. **A**: group ND; **B**: group HFD; **C**: group NETA; **D**: group PD; **E**: group NETA + PD

Upon inspection, liver in HFD, NETA, PD and NETA + PD groups appeared to be enlarged with substantial deposition of adipose tissue around the abdominal region compared to group ND, with group HFD and NETA + PD the most prominent. As H&E staining showed, histological analysis of the hepatic fragments in group ND exhibited well-preserved architecture with characteristic hepatocytes distributed homogenously throughout the hepatic parenchyma. The other 4 groups exhibited alterations, including tissue disorganization with both microvesicular and macrovesicular steatosis in the cytoplasm of the hepatocytes, as well as the presence of multiple foci of inflammable infiltrates, as demonstrated in Fig. 2A, 2B, 2C, 2D, 2E.

As for degree of steatosis grading, two independent investigators blinded to treatment groups examined the sections under light microscopy. Hepatic steatosis was graded according to the magnitude of steatosis (both macro- and micro-vesicular fat accumulation) as published before [19]. Briefly, the degree of steatosis was graded 0–4 (grading 0 =  < 5%, 1 = 5–25%, 2 = 26–50%, 3 = 51–75%, 4 =  > 75%), based on the average percentage area of the liver section that was occupied by fat vacuoles per field at 100 × magnification under H&E staining in 20 random fields.

Score of steatosis is significantly higher in group HFD, and alleviated in group of NETA + PD compared with group HFD (2.73 ± 0.65 vs 3.78 ± 0.44 *P* < 0.01). No changes were found between other groups (Fig. 2, Table 1).

The results showed that α-NETA and α-NETA plus PD98059 improved hepatic steatosis but no impact on TNF-α level of liver homogenates, infers that there was no influence on the progression of steatosis to steatohepatitis.

### α-NETA and α-NETA plus PD98059 improved biochemical determinations

The serum levels of ALT, AST, TC, and TG in different groups are demonstrated in Table 2.

**Table 2:** Serum ALT, AST, TC and TG levels

Group	ALT (U/L)	AST (U/L)	TC (mmol/L)	TG (mmol/L)
Group ND	57.00 ± 9.17 ^b^	222.67 ± 22.55 ^a^	3.17 ± 0.88 ^a^	1.36 ± 0.09 ^b^
Group NETA	144.76 ± 19.18	200.40 ± 73.26 ^a^	8.29 ± 1.47	1.05 ± 0.17
Group PD	171.70 ± 89.29	468.30 ± 37.30	6.93 ± 0.13	1.08 ± 0.13
Group NETA + PD	79.59 ± 30.13 ^a^	170.41 ± 56.78 ^a^	7.58 ± 0.16	1.18 ± 0.02
Group HFD	202.00 ± 54.99	315.67 ± 25.70	6.83 ± 0.41	1.18 ± 0.02

*ALT* Alanine aminotransferase, *AST* Aspartate aminotransferase, *TC* Total cholesterol, *TG* Triglyceride

^a^*P* < 0.05 vs group HFD

^b^*P* < 0.01 vs group HFD

Table 2 shows ALT, AST, TC and TG levels were increased in group HFD compared with group ND. AST levels decreased in group of NETA and NETA + PD, while ALT declined only in group of NETA + PD compared with group HFD.

### α-NETA down-regulated elevated mRNA expressions of CMKLR1 in ileal tissues

**Fig. 3:**
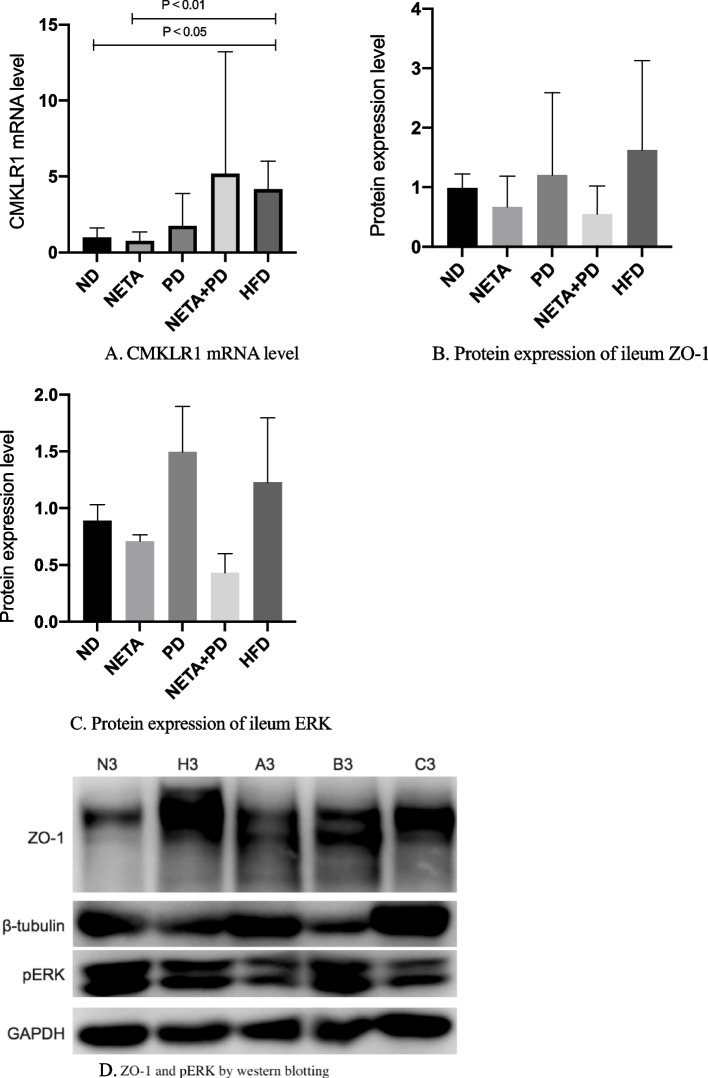
**A** shows the mRNA expression of CMKLR1 was significantly higher in ileums in group HFD (4.17 ± 1.84 vs 1.00 ± 0.62, *P* = 0.047), and down-regulated by α-NETA (0.75 ± 0.61 vs 4.17 ± 1.84, *P* = 0.007). Fig **B**, **C**, **D** show no differences of ZO-1 or pERK1/2 were detected between groups by western blotting. N: group ND; H: group HFD; A: group NETA; B: group PD; C: group NETA + PD

The mRNA expressions of CMKLR1 in ileums of group ND, group NETA, group of PD, group of NETA + PD, and group of HFD were shown in Table 3. Compared with group ND, the mRNA expression of CMKLR1 was significantly higher in ileums in group HFD (4.17 ± 1.84 vs 1.00 ± 0.62, *P* = 0.047), and down-regulated by α-NETA (0.75 ± 0.61 vs 4.17 ± 1.84, *P* = 0.007, Table 3. Figure 3A).

**Table 3:** CMKLR1, ZO-1 and pERK expressions in ileal tissues of group ND, group NETA, group PD, group NETA + PD, and group HFD

Gene	Group	mRNA level^a^	Protein level^b^
CMKLR1	Group ND	1.00 ± 0.62	No detectable
	Group NETA	0.75 ± 0.61^d^	No detectable
	Group PD	1.76 ± 2.12	No detectable
	Group NETA + PD	5.18 ± 8.03	No detectable
	Group HFD	4.17 ± 1.84^c^	No detectable
ZO-1	Group ND		0.99 ± 0.24
	Group NETA		0.67 ± 0.52
	Group PD		1.21 ± 1.38
	Group NETA + PD		0.55 ± 0.47
	Group HFD		1.63 ± 1.50
pERK	Group ND		0.79 ± 0.39
	Group NETA		0.75 ± 0.38
	Group PD		1.78 ± 0.88
	Group NETA + PD		0.31 ± 0.08
	Group HFD		0.83 ± 0.34

^a^mRNA abundance was analyzed using the 2-^ΔΔct^ Ct method with GAPDH as the constitutive marker

^b^The protein expression was analyzed using integrated intensity with β-actin as the constitutive marker

^c^*P* < 0.05 compared with group ND

^d^*P* < 0.01 compared with group HFD

### α-NETA had no impact on protein expressions of ZO-1 and pERK in ileal tissues by western blotting

Protein expressions of ZO-1 and pERK1/2 were analyzed through western blotting on the ileum samples. There are no differences of protein expression of ileal ZO-1 or pERK1/2 between groups. We detected no CMKLR1 protein with western blotting indicating a discrepant mRNA and protein expression (Table 3. Figure 3B, 3C, 3D).

### α-NETA had no impact on endotoxin levels

Serum endotoxin levels are illuminated in Table 1. As Table 1 show: endotoxin level in group HFD is higher than in group NETA and NETA + PD, but failed to attain significant differences (*P* = 0.067, comparing group HFD with group NETA + PD; *P* = 0.093, comparing group HFD with group NETA + PD), indicating that there is no evidence to support the hypothesis that α-NETA interacts with gut microbiota, influences intestinal mucosal integrity, hence to alleviate the progression of hepatic steatosis.

## Discussion

In this study, we demonstrate that mRNA of CMKLR1 is upregulated in ileal tissues of HFD-induced obese mice which can be revised by CMKLR1 Inhibitor α-NETA. We find that α-NETA prevents body weight gains and further enhanced by ERK inhibitor PD98059. We find steatosis only alleviated in mice with less weight gain, indicating that α-NETA exerts its weight gains modulating effect in turn to alleviate steatosis, but no direct impact on intestinal mucosa integrity and alleviation of steatohepatitis.

Since there is no effective drug for NAFLD, exploring potential therapeutic strategies for NAFLD or NASH is of great importance and urgently needed. In our previous study, we reported that HCBP6 is involved in the development of steatosis with which we suggested that HCBP6 could emerge as a marker in NAFLD development [20]. In recent years, apart from that obesity and a combination of genetic, metabolic, lifestyle, as well as environmental factors have been confirmed to be related with NAFLD [21, 22]. Studies have revealed that intestinal factors are relevant to NAFLD process [23]. In NAFLD, the disorder of microbes and enhanced intestinal permeability expose the liver to enteric-derived bacterial metabolites, leading to chronic endotoxemia and related change in the gut − liver axis, suggesting that the inflammatory or cytokines markers in the small intestine could serve as the target in NAFLD treatment [24]. A study by Zhao [25] revealed that berberine, which is widely used in china to combat intestine inflammations, exerts improving effect on glucogenesis and lipid metabolism in nonalcoholic fatty liver disease. In the present study, we focus on CMKLR1 in the small intestine.

CMKLR1 is G protein-coupled receptor that binds chemerin, a proteolytically regulated leukocyte chemoattractant identified in 2003 as the product of the RARRES2 gene [26, 27]. Chemerin/CMKLR1 plays important roles in inflammation, chemotaxis of immune cells, as well as in metabolic syndrome [28–30]. Chemerin and its receptors are found abundantly expressed in adipose tissues and liver tissues, but a detailed role of chemerin in hepatic function and metabolic liver diseases has not yet been clearly explored [27, 31, 32]. Liu et al. [10] demonstrated that the lentivirus mediated CMKLR1 over expression in adipose tissue of rats can significantly improve the nonalcoholic fatty hepatitis liver tissue pathology, while Zhang [12] reported that the increased expression of CMKLR1 may aggravate liver damage. Since severities of liver disease may influence chemerin/CMKLR1 axis in the liver tissues [32], we believe that investigation on guts may help to get insight the role of chemerin/CMKLRS axis on NAFLD.

The expression of CMKLR1 in small intestine has rarely been reported. Initial studies revealed that the RvE1-receptor (ChemR23 or CMKLR1) is expressed on intestinal epithelial cells and correlated with regulation of inflammatory response gene expression. A report by Eri [33] indicated that Resolvin-E1 elicits an epithelial resolution signature through RvE1-receptor, further induced expression of intestinal alkaline phosphatase (ALPI) which has been showed to detoxified bacterial LPS. A study by HJ [34] demonstrated that CMKLR1 knockout mice exhibited decreased abundance of *Akkermansia* and *Prevotella*, with which a negative relationship was found only significant with total body weight. In our study, we found that the mRNA expression was increased in the ileum of the obese steatotic mice. Furthermore, we found α-NETA, a specific CMKLR1 antagonist, decreased the CMKLR1 expression and exerted its weight modulating effect. Although we have not investigated the changes of the gut flora, our result is consistent with report by HJ [34], who reported that CMKLR1 KO is benefit for HFD induced metabolic diseases. We have noticed hepatic steatosis only was alleviated in mice with less weight gain, consistent with the study by Xue et al., who demonstrated that α-NETA inhibits fat deposition in the liver and adipose tissue as well as lipid accumulation in the liver of high-fat fed mice [7].

To determine whether the expression of CMKLR1 is related to the permeability of the small intestine, we check the expression of ZO-1, a cytoplasmic-protein members of the membrane-associated guanylate kinase family of proteins [35]. We found no impact of α-NETA on ileal ZO-1 expression and no significant changes of serum endotoxin levels. The alleviating effect on steatosis are only notified in mice with less weight gain, indicating that there is no sufficient evidence to reveal that intestinal chemerin/CMKLR1 pathway is involved in gut-liver axis hence the progress of NAFLD.

MAPK is an intracellular serine/threonine protein kinase involved in transduction of extracellular stimulation to the cell and its nucleus. MAPK consists of ERK, c-Jun N-terminal kinase (JNK)/stress-activated protein kinase (SAPK) and p38 [36]. ERK pathway has been reported to be correlated with obesity and insulin resistance [37]. Peng et al. [38] reported that CMKLR1 expression was upregulated in pulmonary arterial smooth muscle cells (PASMCs) in response to hypoxia or chemerin stimulation. Notably, the author noted that the regulatory effects of chemerin on PASMCs were blunted by PD98059, demonstrating the relation of ERK pathway with chemerin/CMKLR1 axis. In our present study, we found that α-NETA reduced body weight enhanced by PD98059, inferring a synergetic effect in blocking chemerin/CMKLR1 axis and ERK pathway. However, we found no impact of α-NETA on pERK in ileal tissues detected by western blotting, demonstrating that the cross talk between chemerin/CMKLR1and ERP pathway remains to be verified.

In conclusion, we demonstrate that CMKLR1 is upregulated in ileal tissues of HFD-induced obese mice which can be reversed by CMKLR1 inhibitor α-NETA. α-NETA prevented body weight gain further enhanced by ERK inhibitor PD98059, but no impact of α-NETA on ZO-1 or pERK in ileal tissues was found, indicating that there is no evidence to confirm whether the intestinal Chemerin/CMKLR1 axis is involved in intestinal permeability via ERK pathway hence to influence the steatohepatitis progression.

## Supplementary Information

**Additional file 1.**

**Additional file 2.**

**Additional file 3.**

**Additional file 4.**

## Data Availability

The datasets used and/or analyzed during the current study are available from the corresponding author on reasonable request.
