# Supplementary figures and images for: α-NETA down-regulates CMKLR1 mRNA expression in ileum and prevents body weight gains collaborating with ERK inhibitor PD98059 in turn to alleviate hepatic steatosis in HFD-induced obese mice but no impact on ileal mucosal integrity and steatohepatitis progression
Source: BMC Endocr Disord. 2023 Jan 10;23:9. doi: 10.1186/s12902-023-01267-9 (PMC9830776; doi:10.1186/s12902-023-01267-9)

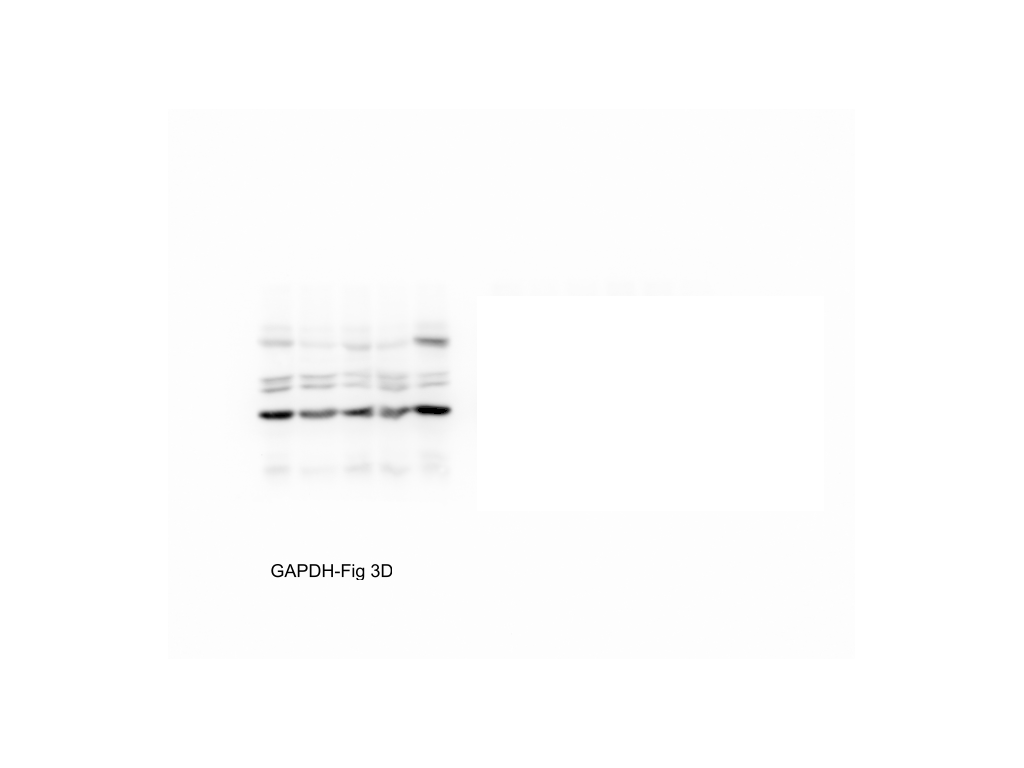

Supplement: Supplementary file 1 — Additional file 1. [file 12902_2023_1267_MOESM1_ESM.tiff]

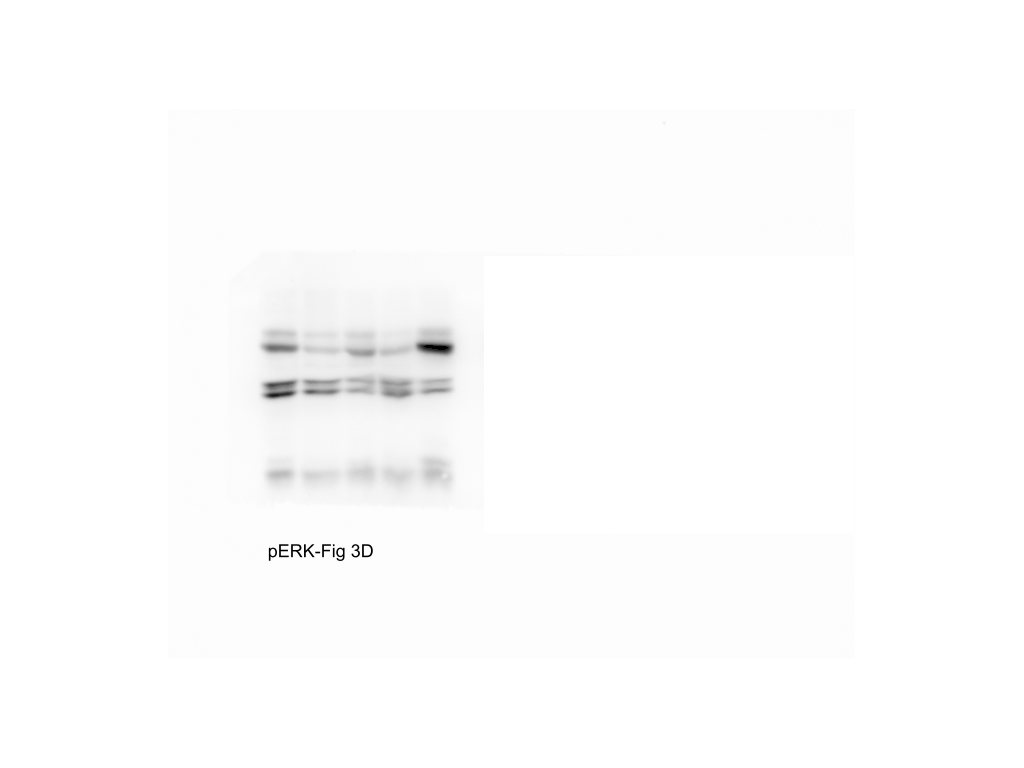

Supplement: Supplementary file 2 — Additional file 2. [file 12902_2023_1267_MOESM2_ESM.tiff]

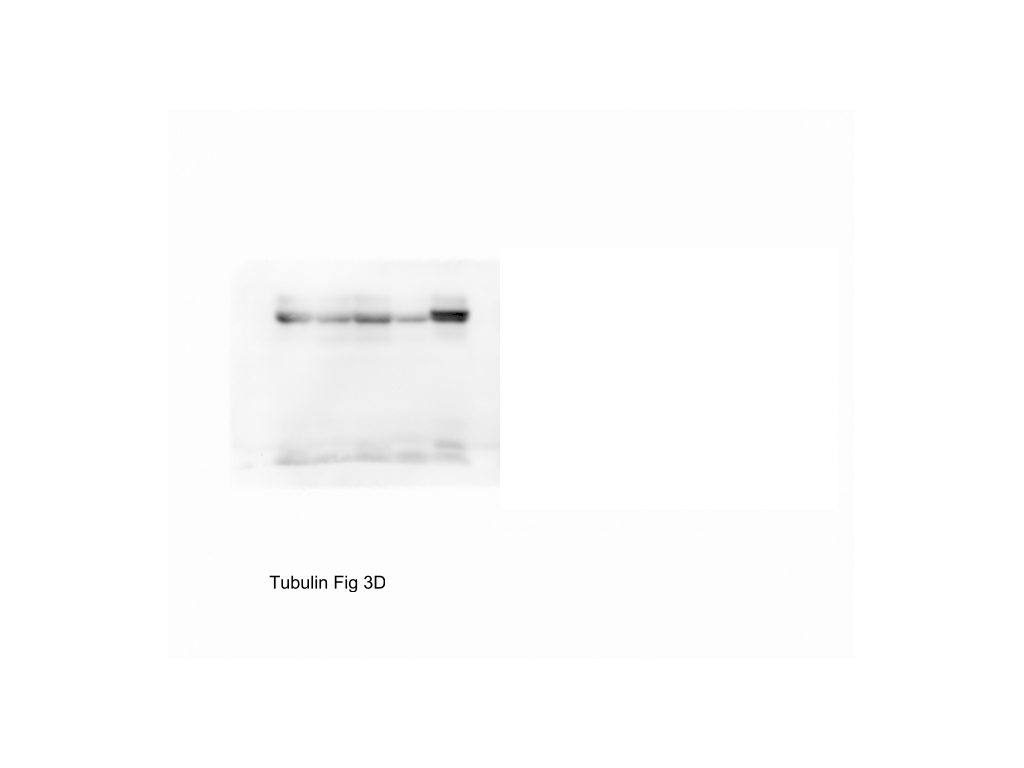

Supplement: Supplementary file 3 — Additional file 3. [file 12902_2023_1267_MOESM3_ESM.tiff]

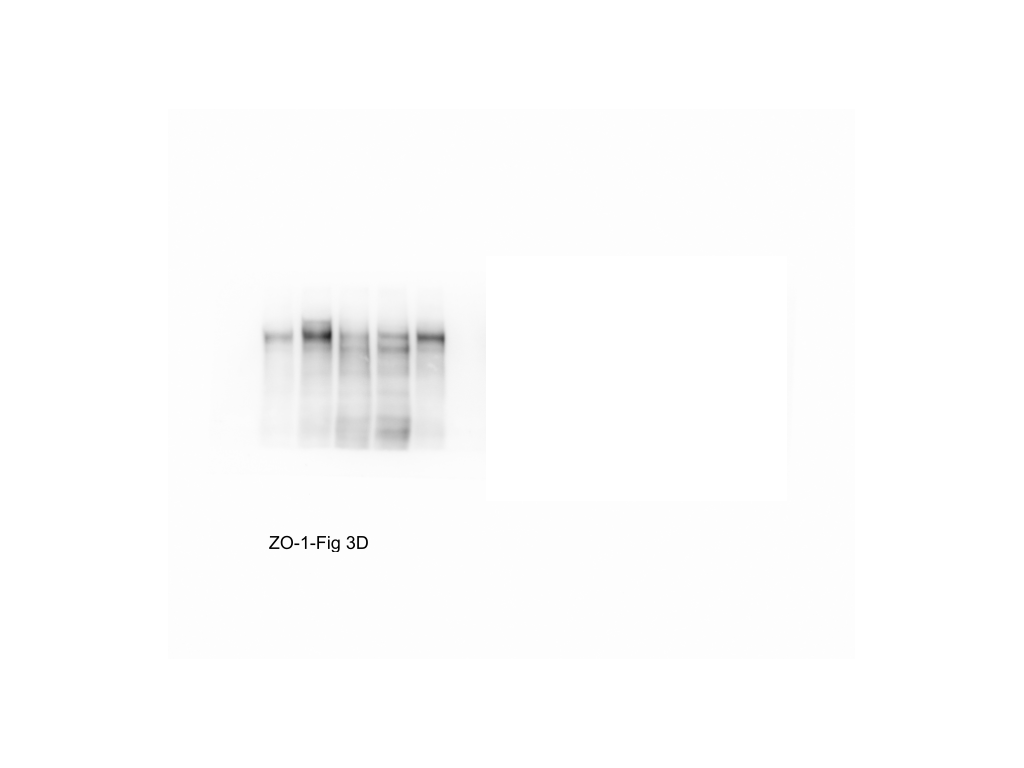

Supplement: Supplementary file 4 — Additional file 4. [file 12902_2023_1267_MOESM4_ESM.tiff]
